# Should the Left Atrial Appendage Closure (LAAC) Technique Be the Main Form of Stroke Prevention in Patients With Long-Standing Persistent or Permanent Atrial Fibrillation?

**DOI:** 10.7759/cureus.54256

**Published:** 2024-02-15

**Authors:** Thebuoshon Amalathasan, Pooja A Nagaratnam, Mirna El Dirani, Julius M Nagaratnam, Samer Kholoki

**Affiliations:** 1 Internal Medicine, All Saints University School of Medicine, Chicago, USA; 2 General Medicine, Bond University, Gold Coast, AUS; 3 Internal Medicine, Saint James School of Medicine, Chicago, USA; 4 Internal Medicine, Avalon University School of Medicine, Phoenix, USA; 5 Internal Medicine, La Grange Memorial Hospital, Chicago, USA

**Keywords:** permanent afib, long-standing afib, stroke, left atrial appendage closure (laac), atrial fib

## Abstract

Currently, oral anticoagulants are considered the gold standard for stroke prevention in patients with atrial fibrillation. Despite the efficacy of oral anticoagulants in reducing stroke incidence, patients are at risk of developing adverse reactions such as excessive bleeding and bruising, and can also have drug-drug interactions. In the early 2000s, a minimally invasive technique called the left atrial appendage closure emerged as an alternative for stroke prevention in atrial fibrillation patients who could not tolerate oral anticoagulants. Despite the success of the left atrial appendage closure, practitioners still opt for medication therapy and are reluctant to advocate for this procedure. Given the adverse effects of oral anticoagulants, physicians should question if this is the appropriate method of stroke prevention in long-standing persistent or permanent atrial fibrillation patients. This case report investigates an 82-year-old Middle Eastern male in the United States with long-standing persistent atrial fibrillation who underwent a left atrial appendage closure due to recurrent bleeding on oral anticoagulants. In addition, there will be further discussion on the appropriate method of stroke prevention in similar patients.

## Introduction

Atrial fibrillation (Afib) is an irregular rhythm that affects the ability of the atria to contract efficiently and increases the risk of thromboembolic events like a stroke [1]. Afib patients are further categorized into paroxysmal, persistent, long-standing persistent, and permanent Afib [2]. Per the 2023 American College of Cardiology (ACC)/American Heart Association (AHA) guidelines, paroxysmal Afib is when patients have intermittent Afib episodes that terminate within seven days of onset [2]; persistent Afib is when patients have sustained Afib episodes for greater than seven days and require intervention [2]; long-standing persistent Afib is when patients have continuous Afib for greater than twelve months [2]; and permanent Afib is when no further attempts are made at rhythm control by the patient or physician [2].

A patient’s risk of developing a stroke is determined using the CHA2DS2VASC score [3]; one-point for (C)ongestive heart failure (CHF), one-point for (H)ypertension (HTN), one-point for (A)ges between 65-74-years or two-points for ages greater than or equal to 75-years, one-point for (D)iabetes, two-points for (S)troke or transient ischemic attacks, one-point for female sex, and one-point for (VASC)ulopathies [3]. A study conducted in 2013 using the Anticoagulation and Risk Factors in Atrial Fibrillation (ATRIA) cohort [4], determined the risk of stroke with varying CHA2DS2VASC scores, which were later published in the 2023 ACC/AHA guidelines [2,4]. Patients with one point had a stroke risk of 0.55 per 100-person-years [2,4]; patients with two points had a stroke risk of 0.83 per 100 person-years [2,4]; and patients with scores ranging between three points and nine points, had a stroke risk ranging between 1.66 and 16.62 per 100 person-years respectively [2,4].

Due to the risk of stroke in Afib patients, physicians are inclined to provide oral anticoagulants (OAC) such as apixaban *(branded Eliquis)*, dabigatran *(branded Pradaxa)*, rivaroxaban *(branded Xarelto)*, and warfarin for stroke prevention [5]. Currently for patients who are intolerant to OAC due to reasons such as severe bleeding, medication and dietary interactions, and cost restrictions, a procedure known as the transcatheter left atrial appendage closure (LAAC), first developed in 2001-02 [6], is available as an alternative for stroke prevention. The procedure is used to occlude the left atrial appendage (LAA), the site of 90% of thrombus formation in Afib patients [7]. The two current commercially available LAAC devices in the United States (US) include the Boston Scientific WATCHMAN FLX LAAC device (Boston Scientific, Marlborough, USA), and the Abbott Amplatzer Amulet LAA occluder device (Abbott, Chicago, USA) [8].

Currently, OAC is considered the gold standard for stroke prevention in Afib patients [9]. However, physicians should acknowledge the adverse effects of OAC in patients who would require long-term therapy and consider the alternative transcatheter LAAC.

## Case presentation

The patient is an 82-year-old Middle Eastern male residing in Chicago, US. Prior to his arrival in the US, he was residing in Egypt, where he had an acute inferior myocardial infarction (MI) in October 2006. His MI was complicated with a cardiac arrest secondary to Torsade De Pointes requiring cardiopulmonary resuscitation, defibrillation and epinephrine infusion to regain consciousness. He had a left heart catheterization (LHC) with deployment of a bare metal stent in the distal right coronary artery (RCA). A subsequent LHC in September 2008 in the US resulted in the deployment of a drug-eluting stent in the proximal first diagonal (D1) branch of the left anterior descending (LAD) artery. The patient had subsequent surveillance LHC in the US on 21st July 2009, 15th July 2011, and most recently on 18th October 2022 which showed a 20% distal left main artery stenosis, patent LAD, visible stent in D1 with mild in-stent restenosis, 30-40% mid left circumflex stenosis, and a 20% proximal and 30-40% distal RCA stenosis. Prior RCA stent was not noted in the LHC.

The patient was first diagnosed with Afib in 2020, which responded appropriately to rate control therapy of metoprolol succinate 50 mg once daily (QD), however, it failed to convert to sinus rhythm on antiarrhythmic therapy of dronedarone 400 mg twice daily (BID). On 12th December 2022, the patient had a discussion with an electrophysiologist (EP) regarding candidacy for an atrioventricular nodal (AVN) ablation with a permanent pacemaker implantation. He was notified that the procedure may eventually be required due to difficulty in rate control with AVN blocking medications given he usually has a blood pressure on the lower limit of normal. The patient was ruled as having long-standing persistent Afib after multiple office electrocardiograms (ECG) showed Afib with a controlled ventricular rate (CVR), and a Zio Patch heart monitor (iRhythm Technologies, San Francisco, USA) completed on 20th March 2023 showed a 100% Afib burden (average rate of 78 beats per minute (bpm), range: 46-179bpm) and a 6.4% burden of premature ventricular contractions. The patient had an additional past medical history of HTN, hyperlipidemia, diastolic CHF, mild stenosis of the bilateral internal carotids (16-49% stenosis on a carotid ultrasound done 6th April 2023), valvulopathies of mild mitral regurgitation (MR) and moderate tricuspid regurgitation ((TR) on a transesophageal echocardiogram (TEE) done 17th April 2023), chronic obstructive pulmonary disease and prostate cancer (diagnosed 2016). He had a CHA2DS2VASC score of five points and was placed on rivaroxaban 20 mg QD for stroke prevention.

In December 2020, the patient was complaining of having melena and was eventually referred to a gastroenterologist. A colonoscopy on 2nd February 2021 showed two polyps in the mid-ascending and distal transverse colon, measuring 0.5 cm and 0.7 cm, respectively, that were removed by cold biopsy and cold snare polypectomy. Pathology reports showed that the polyps were tubular adenomas, and the patient’s melena was ruled as being of upper gastrointestinal etiology. The patient’s hemoglobin (Hgb) levels declined from 13.9 g/dL on 27th October 2020 to 9.2 g/dL on 8th February 2021, and his rivaroxaban was discontinued. His Hgb levels then increased to 15.1 g/dL on 11th May 2021 with additional iron supplementation, remaining within normal limits on subsequent labs. His rivaroxaban regimen was re-started on 24th January 2023, however, he had episodes of hematuria, causing his Hgb to decrease to 12.9 g/dL on 9th March 2023. 

**Figure 1:**
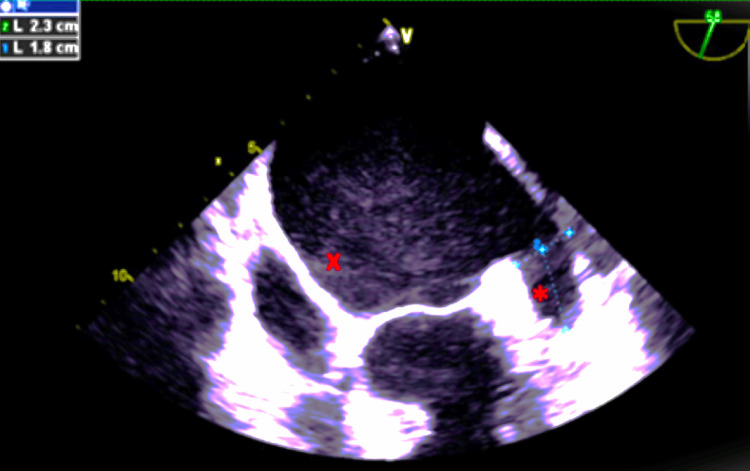
Pre-LAAC transesophageal echocardiogram at 68 degrees. * = left atrial appendage; X = left atrium; LAAC = Left Atrial Appendage Closure

Due to his recurrent bleeding on rivaroxaban, an EPwas consulted for an LAAC as an alternative for stroke prevention and was ruled as an appropriate candidate. A pre-procedural TEE on 17th April 2023 showed normal ejection fraction (EF), mild MR, moderate TR, no thrombus in the left atrium (LA) or LAA, and the LAA measuring 1.7 cm x 2.4 cm at 46°, 1.8 cm x 2.3 cm at 68° (Figure 1), 1.8 cm x 2.1 cm at 133° and maximum dimensions of 1.7 cm x 2.4 cm.

**Figure 2:**
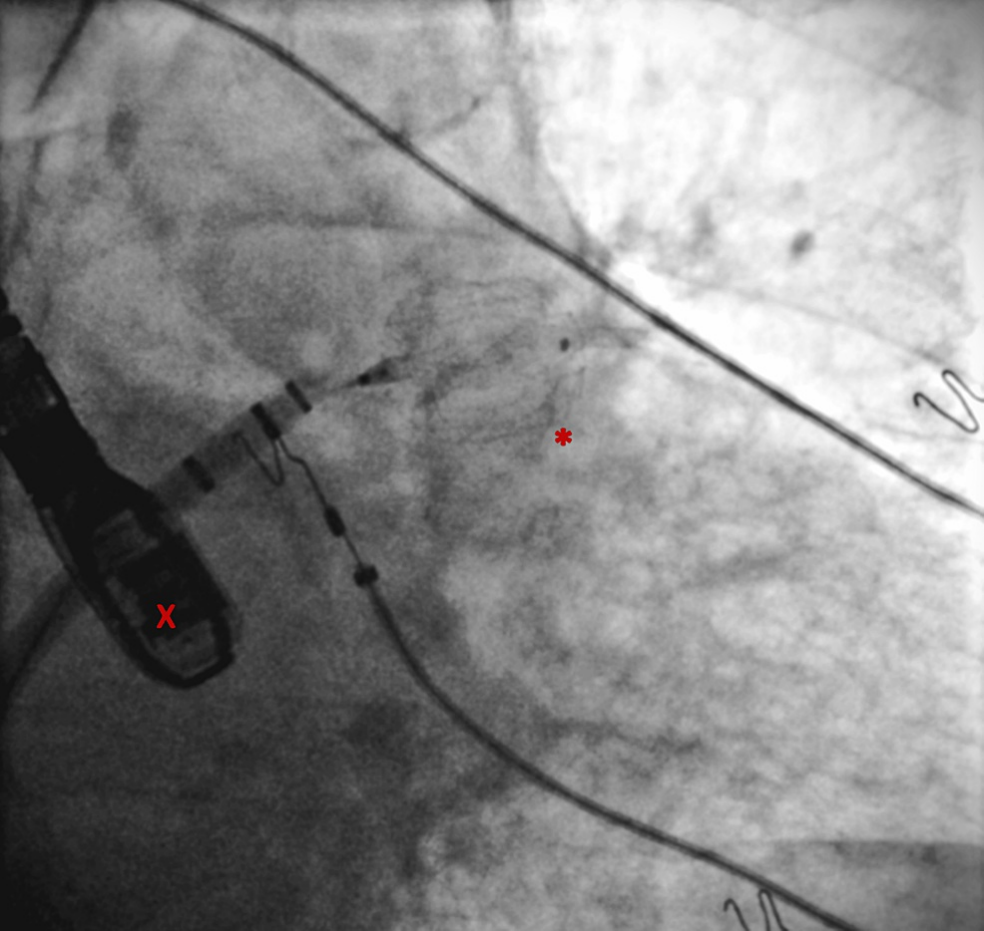
27 mm Watchman FLX being deployed after transseptal access, with TEE and X-ray fluoroscopy guidance. * = Deployed Watchman FLX; X = TEE (transesophageal echocardiogram)

**Figure 3:**
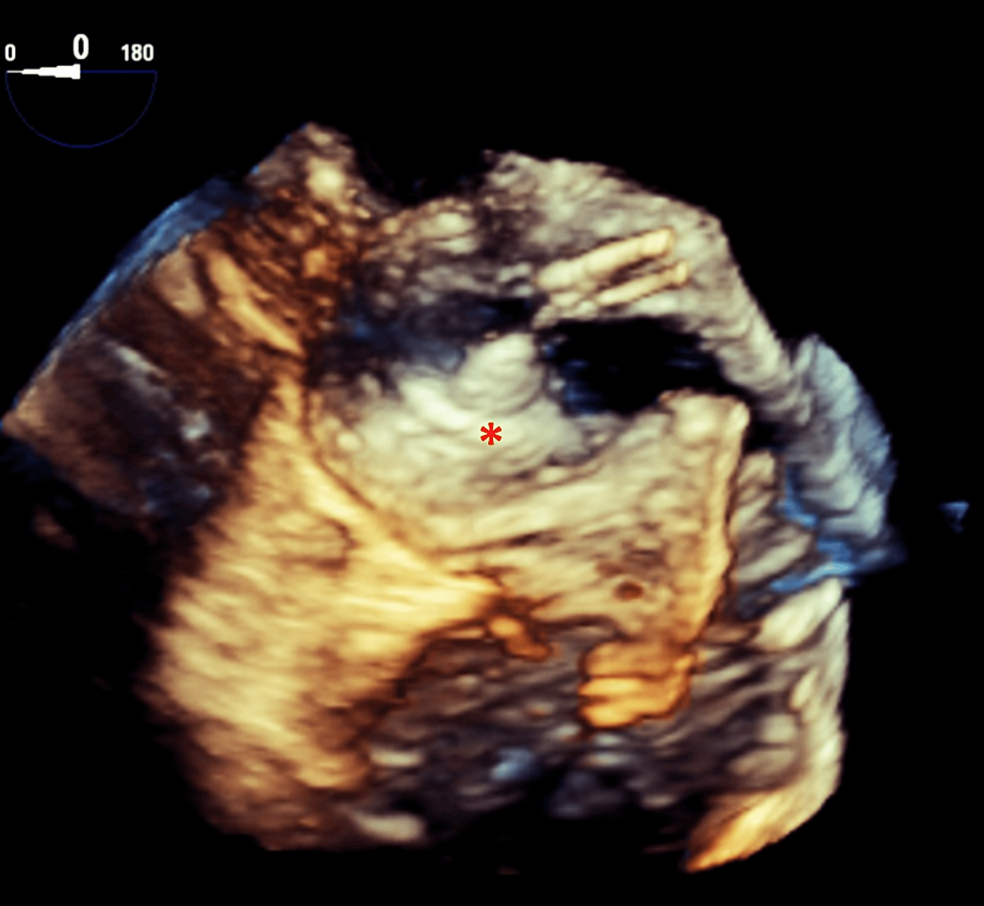
Transesophageal echocardiogram showing 27 mm Watchman FLX positioned in the LAA (surgeons view). * = 27 mm Watchman FLX occluding the left atrial appendage

A 27 mm Watchman FLX device (Boston Scientific, Marlborough, USA) was deployed in the LAA on 9th May 2023 via femoral vein access, transition across the interatrial septum (Figure 2), and 30-40% compression. A post-procedural TEE on 9th May 2023 showed normal EF, a well-seated Watchman in the LAA on tug test (Figure 3), a residual atrial shunt post-transseptal catheterization, no device-related thrombus or peri-device leaks, and no pericardial effusion.

**Figure 4:**
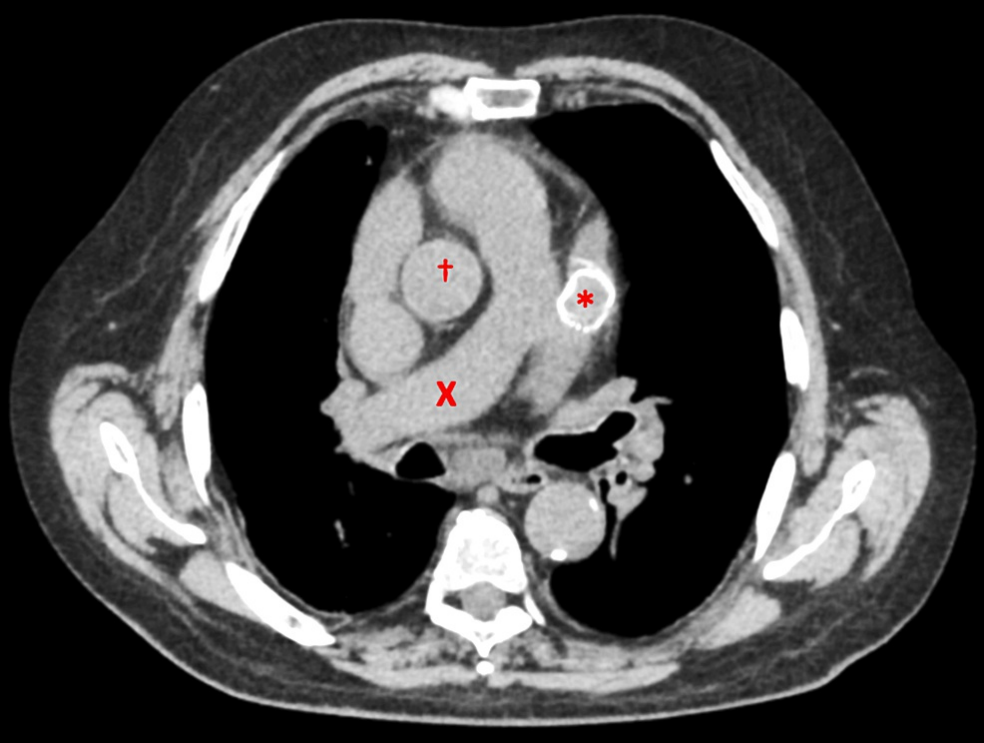
Chest CT showing the Watchman FLX positioned in the left atrial appendage. * = 27 mm Watchman FLX; X = pulmonary artery; † = aorta

On postop day five, the patient readmitted himself for complaints of pleuritic chest pain and dyspnea. ECG showed Afib with CVR and no ischemic changes. High-sensitivity troponins were 11 ng/dL, and brain natriuretic peptide was 332 pg/mL. Acute coronary syndrome was ruled out, and the patient was transferred to the telemetry ward with diuresis on intravenous furosemide 20 mg every 12 hours. Chest computerized tomography (CT) on 15th May 2023 showed 7-8 mm nodular calcifications in the right lobe of the thyroid, a 3.4 cm main pulmonary artery, mild cardiomegaly, a device in the LAA (Figure 4), a 7-8 mm nodule in the right-middle lobe of the lung, and a 6 mm nodule in the right upper lobe of the lung. The patient had a sedimentation rate of 43 mm/hour and was started on colchicine 0.6 mg BID which improved his symptoms. The patient was transitioned to oral furosemide 20 mg BID and discharged on 18th May 2023.

Six-week post-Watchman TEE done on 23rd June 2023 showed an EF of 55%, well-seated Watchman in the LAA without device-related thrombus or peri-device leaks, an iatrogenic patent foramen ovale with predominant left-to-right shunting, and no pericardial effusion. The patient’s rivaroxaban was subsequently discontinued and then placed on dual antiplatelet therapy (DAPT) of clopidogrel 75 mg QD and aspirin 81 mg QD to prevent device-related thrombus formation.

## Discussion

This case clearly highlighted the challenges of OAC that physicians should consider when treating patients with long-standing persistent Afib. A high bleeding risk is one of the main adverse effects of long-term OAC [10] as evidenced in this patient, and is prevalent in the setting of comorbidities [10]. A patient’s risk of bleeding on OAC is determined by the HAS-BLED score [11], which is calculated using factors such as (H)TN, (A)bnormal renal or liver function levels, (S)troke, (B)leeding history or a predisposition to bleeding events, (L)abile Internationalized Normalized Ratio (INR, not within the therapeutic range less than 60% time), (E)lderly patients (age greater than 65-years), and the concomitant use of (D)rugs and alcohol [11]. Patients with a score of zero points are at low risk of bleeding; scores of one or two points suggest a moderate risk of bleeding; and scores of three points or more suggest a high risk of bleeding [12]. 

Prior studies have established a significant association (p=0.03) between major bleeding events and patients of advanced age (greater than 75 years) requiring OAC for stroke prevention [13]. Prior studies have also established a positive association between age and comorbidities such as HTN, and liver and kidney disease [14,15], which further increases the bleeding risk. The LAAC aims to reduce the risk of Afib-related strokes by occluding the LAA, the primary site of thrombus formation in Afib patients [7], allowing patients to transition off OAC, mitigating the risk of bleeding. It is true that after the LAAC, patients are required to be on DAPT for six months to prevent device-related thrombus formation, which carries some risk of bleeding. However, a prior study assessing 298 patients on DAPT after LAAC showed only 4.4% of patients had non-procedure-related bleeding [16]. The risk of bleeding on DAPT post-LAAC is minimal, and the benefits of the procedure outweigh the risks, making it the more favorable option for stroke prevention.

Additionally, in patients with long-standing persistent and permanent Afib, there is difficulty converting them to sinus rhythm (SR) via medication therapy or minimally invasive procedures. Physicians opt for procedures such as TEE-guided direct current cardioversion (DCCV), and a transcatheter Afib ablation to convert patients to SR when medication therapy is unsuccessful. However, prior studies predicted DCCV to be successful in patients in Afib for less than six months (p<0.04, odds ratio [OR] 2.2, 95% confidence interval [CI]: 1.1-4.6) [17]. Additionally, the maintenance of SR post-DCCV was predicted in patients in Afib for less than three months (p<0.04, OR 2.5, 95% CI: 1.1-5.6) [18]. A transcatheter Afib ablation is also found to be less successful in patients with non-paroxysmal Afib [18]. A prior systematic review and meta-analysis reviewing seventeen studies showed a 41.8% success rate (95% CI 25.2%-60.5%) after a single ablation in non-paroxysmal patients in studies with a follow-up greater than three years [18]. Since patients in long-standing persistent and permanent Afib have a low success rate to convert to SR, the LAAC should be considered for long-term stroke prevention to mitigate the chronic use of OAC.

Despite the success of the LAAC, the procedure is underutilized with physicians usually opting for OAC therapy as the primary method of stroke prevention. This reluctance can be attributed to the LAAC being a minimally invasive procedure with an associated risk for bleeding, device-related thrombus, peri-device leaks, cardiovascular events, and stroke [19]. A prior prospective study following 139 patients five years after the LAAC showed that the occurrence of these post-procedural complications was very uncommon [18]. Of the 139 patients, 3.6% had a peri-device leak of 1 mm to 3 mm, 3.1% had an episode of severe bleeding, 1.6% had a thrombus in the LA, 3.9% had a cardiovascular event, and 0.8% had a stroke within five years of the LAAC [19]. Given the low percentage of post-procedural complications, physicians should be more confident in endorsing long-standing persistent and permanent Afib patients for the LAAC. Understandably, physicians should also ensure that patients are able to tolerate the demands of general anesthesia, and the use of iodine contrast and x-ray fluoroscopy during the LAAC.

Another factor that could explain the lack of appeal for the LAAC procedure by physicians and patients, is the cost of the procedure. A prior study in 2015 assessed the cost-effectiveness of the LAAC as opposed to OAC using data from the Centers for Medicare and Medicaid Services [20]. The study showed that the LAAC was found to be more cost-effective than warfarin at seven years, and non-warfarin OAC at five years [20]. At the ten-year mark, LAAC was found to provide more quality-adjusted life-years than both warfarin and non-warfarin OAC [20]. Even if the cost of the LAAC was doubled, the study showed that the procedure was more cost-effective than both warfarin and non-warfarin OAC at the eleven-year and ten-year mark respectively [20]. Given the cost-effectiveness of the LAAC compared to OAC, physicians should advocate for the most financially optimal approach for stroke prevention in patients with long-standing persistent and permanent Afib, especially in the setting of recurrent bleeding.

## Conclusions

The LAAC procedure is a valuable alternative in stroke prevention for patients with long-standing persistent and permanent Afib. Given the high risk of bleeding on OAC with increasing age and the lack of success in converting long-standing persistent and permanent Afib patients to SR, physicians should demonstrate more confidence in recommending the procedure. Physicians should also advocate for the LAAC since it has been demonstrated to be more cost-effective in the long term as opposed to OAC. By transitioning patients off OAC, physicians are able to better optimize the medical management of patients, whilst providing adequate stroke protection. Further physician education would be beneficial in improving the advocacy of the LAAC in long-standing persistent and permanent Afib patients.
